# Update on *Blastocystis*: highlights from the Fourth International *Blastocystis* Conference

**DOI:** 10.12688/openreseurope.19168.2

**Published:** 2025-03-07

**Authors:** Ana M. Figueiredo, Daisy Shaw, Varol Tunali, Eleni Gentekaki, Anastasios D. Tsaousis, David Carmena

**Affiliations:** 1 Department of Biology and CESAM, University of Aveiro, Aveiro, Portugal; 2 School of Natural Sciences, University of Kent, Canterbury, UK; 3 Department of Parasitology, Celal Bayar University, Manisa, Turkey; 4 Department of Microbiology, Faculty of Medicine, Izmir University of Economics, Izmir, Turkey; 5 Department of Veterinary Medicine, University of Nicosia School of Veterinary Medicine, Nicosia, Cyprus; 6 Parasitology Reference and Research Laboratory, Spanish National Centre for Microbiology, Health Institute Carlos III, Majadahonda, Spain; 7 Centre for Biomedical Research Network in Infectious Diseases (CIBERINFEC), Health Institute Carlos III, Madrid, Spain

**Keywords:** Blastocystis, public health, pathogenicity, evolutionary biology, omics, microbiome, epidemiology, diagnosis, genotyping

## Abstract

While the stramenopile *Blastocystis*, first discovered in 1911, is considered the most prevalent enteric protist in humans, its biology remains largely unexplored. Clinical studies have only recently begun investigating the role of *Blastocystis* in the gut and its relationship with the gut microbiome, and whether it plays a pathogenic role in human and animal health. Aiming to gather leading researchers in the field to encourage and stimulate cross-disciplinary dialogue while fostering long-term international collaborations, the Fourth International *Blastocystis* Conference was hosted from the 17 ^th^ to the 19 ^th^ of September 2024 in Heraklion (Crete, Greece). The event was mainly supported by the COST Action CA21105, “ *Blastocystis* under One Health”, and the Microbiology Society. The multi- and interdisciplinary conference programme covered all aspects related to *Blastocystis* evolutionary biology and advances in omics, intestinal ecology (gut microbiome), clinical significance and association with disease, diagnosis and molecular characterisation, as well as epidemiology and One Health. The high-quality presentations discussed at the conference provided researchers with a synthesis of recent advancements, while key research questions, knowledge gaps, and future steps in *Blastocystis* research were identified. Herein, we aim to provide a thorough overview of the presentations at the conference. The COST Action CA21105, *“Blastocystis* under One Health”, will build on the insights and collaborations fostered during the conference, promoting integrative research approaches, advancing our understanding of *Blastocystis*, and driving future efforts to translate these findings into improved public health strategies.

## Plain language summary

*Blastocystis*, first identified in 1911, is one of the most common intestinal microorganisms found in humans; yet, little is known about its biology. Only recently have scientists started to investigate the role of *Blastocystis* in the gut microbiome and whether it causes disease in humans and animals. The Fourth International *Blastocystis* Conference, which was hosted in Heraklion (Crete, Greece) from the 17 ^th^ to the 19 ^th^ of September 2024, aimed to gather researchers from within the field to advance understanding and foster collaboration. The event was mainly supported by the COST Action CA21105, “ *Blastocystis* under One Health”, and the Microbiology Society. The event brought together research from various fields to discuss topics such as *Blastocystis’* biology, its role in the gut microbiome, links to disease, methods of diagnosis, and its global impact. Presentations highlighted recent advancements in the fields, identified unanswered questions and outlined future research priorities. In this summary, we aim to provide a thorough overview of the presentations at the congress. The COST Action CA21105, *'Blastocystis* under One Health,' will build on the insights and collaborations formed during the conference, to advance our understanding of *Blastocystis* and better understand its implications for public health.

## Introduction

The Fourth International *Blastocystis* Conference (FIBC), co-sponsored by the Microbiology Society and the COST Action CA21105: *Blastocystis* under One Health (see https://www.cost.eu/actions/CA21105/ and https://blastocystis-cost.com/) ^1^, was held in Heraklion, Crete, from the 17 ^th^ to the 18 ^th^ September 2024, with an additional workshop on the 19 ^th^ of September 2024. The conference brought together 51 researchers from 22 countries, ranging from pioneers in the field to early-career scientists, who represent the next generation of researchers in *Blastocystis* research. The conference covered various research areas, including intestinal ecology, clinical significance, evolutionary biology and omics, diagnosis and molecular detection, and epidemiology under the One Health approach. Here, we aim to provide a thorough account of the plenary sessions, invited talks, oral communications, and posters presented at the FIBC, highlighting key presentations and findings from the event, identifying current hot research topics and gaps in knowledge, and outlining future research directions. The original abstracts submitted to FIBC can be found as Extended Data (ED). When referring to them in the text, we have used the following codes: T for Talk and P for Poster. Therefore, T01 would refer to the first talk (either an invited talk or an oral presentation), and P01 would refer to the first poster presentation.

## *Blastocystis* background

*Blastocystis* was first identified in 1911, being reported as *Blastocystis enterocola* ^2^. Soon after, an isolate from a human stool sample was named *Blastocystis hominis* ^3^. Both scientists described it as a yeast. Even though *Blastocystis-*related publications started appearing only a century ago, evidence of the organism’s presence dates back to 595 AD, discovered in paleofaeces ^4^. It was not until 30 years ago that *Blastocystis* was identified as a stramenopile, based on sequencing of the small-subunit ribosomal RNA ( *SSU* rRNA) gene, despite not showing the typical characteristics seen in other members of this clade ^5^.

*Blastocystis* has been found in a broad range of host organisms, including mammals, birds, amphibians ^6^, fish ^7^, reptiles ^8^, and cockroaches ^9^; however, it has not yet been found in plants. The four primary morphological forms of *Blastocystis* are vacuolar, granular, amoeboid, and cyst. Nonetheless, it has been suggested that the granular form of *Blastocystis* might represent degenerative cells in the process of dying, for example, as a result of excessive oxygen exposure ^10– 12^.

Following sequencing of its first complete nuclear *SSU* rRNA gene ^5^, it soon became evident that *Blastocystis* has an extensive genetic diversity, comprising at least seven morphologically identical but genetically distinct organisms ^13^. The development of a simple and robust barcoding method coupled with Sanger sequencing represented a major advance in *Blastocystis* taxonomy ^14^. In 2007, Stensvold *et al*. standardised the *Blastocystis* terminology and introduced the subtype (ST) designation currently in place. Consequently, all mammalian and avian isolates were designated as *Blastocystis* sp. (abandoning the previous *Blastocystis hominis* nomenclature) and assigned to one of nine STs ^15^. Since then, at least 44 *Blastocystis* STs have been documented ^16– 18^, 16 of which (ST1-ST10, ST12, ST14, ST16, ST23, ST35, and ST41) have been found in humans ^19– 22^. Remarkably, nearly one in three reported STs appear to be specific to ruminants, which may act as the main reservoir of the protist. This very same trend might also be applicable to species and ribosomal lineages of the genus *Entamoeba* ^23^.

## Evolutionary biology and advances in Omics

*Blastocystis,* finding its taxonomic home, brought forth the question of the evolutionary path taken from the typical stramenopile biflagellate morphology to a non-flagellated organism. Hence, insights gained by looking into a close relative can shed light on the evolution of *Blastocystis*. *Proteromonas lacertae* is recognised as *Blastocystis’* closest known relative, and as such, its genome was compared to that of *Blastocystis* ^24^. This comparative approach revealed similarities and significant differences. For instance, both microorganisms encode the complete endocytic TSET complex, corresponding to the first incidence observed in the Stramenopila. The genome of *P. lacertae* is significantly larger than that of *Blastocystis,* the latter having undergone reductive evolution and loss of genes associated with motility, peroxisomes and mitochondria. This loss of redundancy corresponds to adaptations to life in the gut ^24^.

Omics is an area of biology that is increasingly being used in a multi-disciplinary manner. Omics techniques include but are not limited to proteomics, genomics, metagenomics, transcriptomics, lipidomics, and metabolomics, and provide a global understanding of an organism's biological and cellular processes at a particular time ^25, 26^. Previous genomic studies have already revealed that lateral gene transfer is a key mechanism for the adaptation of *Blastocystis* to the gut ^27^. Still, few full-length *Blastocystis* genomes are available in public databases to help us further understand this and other evolutionary mechanisms, and even fewer with gene predictions and functional annotations. Eukfinder bioinformatics tool ( https://github.com/Rogerlab/Eukfinder) was developed to retrieve *Blastocystis* genomes based on gut metagenomic data ^28^. Additional omics techniques must be utilised to better understand this under-explored protist and the microenvironment required for its growth, both *in vitro* and *in vivo* ^29^. A multi-omics approach focusing on metagenomics and metabolomics was presented at the FIBC. A pilot study was carried out to compare the microbiome composition and metabolome of xenic cultures of *Blastocystis* ST1-ST9, with the eventual aim of integrating this data alongside metatranscriptomic analysis. At the feature level, ST3 has a significantly different bacterial composition from the rest of the STs studied. Time course analysis showed that, over a period of six days in culture, there was no significant change in microbiome composition for any of the studied STs. The metabolomics analysis revealed that ST1-ST3 have a different metabolic profile from the rest of the studied STs. However, further study of the key metabolic pathways involved is required to elucidate the true meaning of these results (Shaw *et al*., ED T21).

## Intestinal ecology (gut microbiome)

As the most frequently recovered microeukaryote from human faecal samples ^30^, studies on *Blastocystis* and the gut microbiome are on the rise. Many presentations at the FIBC focused on the associations of *Blastocystis* with the rest of the gut microbiome. It was highlighted that microbiome studies frequently focus on the bacterial microbiota of an individual, with the microeukaryotic composition often being overlooked. However, a study conducted in a rural community in Colombia demonstrated that *Blastocystis* carriers were associated with the protist *Entamoeba* and the yeast *Hanseniaspora*, whereas non-carriers were associated with the intestinal hookworm *Ancylostoma* and the yeasts *Malassezia*, *Candida,* and *Saccharomyces*. Intriguingly, gut microbiota in Colombian *Blastocystis*-carriers populations mirrored Western Europeans, despite vastly different environments and diets (Garzon *et al*., ED T01). Most findings link *Blastocystis* presence to a eubiotic gut environment and increased bacterial diversity, and this was shown in multiple presentations (Castañeda Garzon *et al*., ED T01; Kwoji *et al*., ED T05; Tomiak, ED T12; Hubáčková *et al*., ED P05). In contrast, Marangi *et al*. demonstrated its association with dysbiosis (Marangi *et al*., ED T09), while others showed that although *Blastocystis* carriage was associated with high bacterial diversity, it was also inversely associated with specific beneficial taxa. However, these findings were based on the bacteriomes of patients with irritable bowel syndrome (IBS) rather than those of healthy individuals (Kwoji *et al*., ED T05). The use of antibiotics has been shown to influence the presence of *Blastocystis* in the gut, with the study of an IBS patient on a 14-day antibiotic course tested for *Blastocystis* carriage. Being positive for *Blastocystis* at the start of the treatment course, the microorganism becomes absent mid-course, correlating with a decrease in bacterial diversity ^31^; however, *Blastocystis* shows resilience, as it reappears post-treatment once the microbiome diversity recovers ^32^.

The conveyed message from the FIBC was that there is a need for more longitudinal studies looking at *Blastocystis* and the gut microbiome, with the most extensive study presented here lasting two years (Marangi *et al*., ED T09), while others are single time-point analyses. There has also been a study involving continuous monitoring of *Blastocystis* carriage for up to one year, showing the persistence of the same STs and alleles in a group of preschool children in central Spain ^33^. A study focusing on children, an under-studied age group, aims to longitudinally collect data on *Blastocystis* prevalence and microbiota composition in children with immunopathological diseases, including Crohn’s disease, type one diabetes, and juvenile idiopathic arthritis. It has already been shown that children with Crohn’s disease have the lowest incidence of *Blastocystis* at 7.5% (Hubáčková *et al*., ED P05). Another presentation highlighted the largest-scale computational study of *Blastocystis* to date, with metagenomic data from nearly 60,000 individuals from 32 countries being analysed ^34^. It was demonstrated that *Blastocystis* carriage is associated with healthier diets (plant-based and unprocessed), lower body mass index (BMI), and the presence of favourable cardiometabolic markers, such as decreased GlycA and increased high-density lipoprotein (HDL), overall suggesting a beneficial role of *Blastocystis* in the gastrointestinal environment (Piperni *et al*., ED T19). This study has challenged our perception of the clinical significance of *Blastocystis*, providing evidence-based data to propose a paradigm shift in which *Blastocystis* should be seen as a symbiont rather than a pathogen ^35^. Similar results have been shown in a large metagenomic survey conducted in 1,581 faecal samples from apparently healthy dairy cattle in France. The authors demonstrated that bacterial alpha-diversity (the number and relative abundance of taxa in an average sample) was higher in the faecal microbiota of *Blastocystis-*colonised cows than in cows without it. Moreover, *Blastocystis* presence at the heifer stage was associated with a 22 % higher productive longevity without compromising the daily milk yield and a 17.5 % lower carbon contribution per kg of milk produced than in *Blastocystis*-free dairy cows (Audebert *et al*., ED T18). This is the first study providing robust evidence of the beneficial effect of *Blastocystis* carriage in a non-human host globally.

On a similar note, only a few *Blastocystis* studies focus on the zoonotic potential of this microorganism, although previous publications include the demonstration of potential zoonotic transmission of *Blastocystis* between non-human primates (NHPs) and their zookeepers ^36, 37^. These findings were supported at the conference by the presentation of a cross-sectional survey of *Blastocystis* in NHPs, also indicating the zoonotic transmission of certain STs (ST1-ST5, ST7, ST8) between these animals and their caregivers (Brožová *et al*., ED P25). Similarly, *Blastocystis* was linked to contact with household pets and farm animals in high-income countries, finding a higher incidence of carriage in people with frequent contact with farm animals and those who undertake long-distance travel (Pavlicková *et al*., ED P20).

Numerous presentations at the FIBC demonstrated future directions in this field, such as the ongoing Danish epidemiological study of Functional Disorders (DanFunD). Expected results include *Blastocystis* prevalence in Denmark and associations with characteristics of carriers, such as the microbiome, clinical factors, and overall health. The study aims to reshape our understanding of *Blastocystis* and its role in the microbiome using metagenomic pipelines (Tomiak, ED T12).

## Clinical significance and association with disease

Several presentations at the FIBC provided mounting evidence for the beneficial effects of *Blastocystis* on gastrointestinal health, as well as associations with favourable cardiometabolic profiles, such as decreased GlycA and increased HDL levels (Piperni *et al*., ED T19). A higher prevalence of *Blastocystis* was recorded in healthy individuals compared to those with immune-mediated inflammatory diseases in Saudi Arabia (El Badry *et al*., ED T13). At the same time, there was no significant association between *Blastocystis* presence and severe conditions like colorectal cancer in Colombian patients (Hernández-Castro *et al*., ED T10). A Spanish clinical study including 170 *Blastocystis*-positive patients solely infected with the protist revealed that the occurrence of clinical manifestations was not associated with any given ST (Seijas-Pereda *et al*., ED P15) ^38^. These studies underscored the complex role of *Blastocystis* in health and disease.

Molecular investigations presented findings on *Blastocystis*’ adaptive mechanisms, particularly superoxide dismutases (SOD1 and SOD2), which are responsible for the organism withstanding oxidative stress, likely supporting its persistence in the gut microbiota (Denoyelle *et al*., ED T08).

Findings from an experimental colitis model in rats revealed that long-term colonisation with *Blastocystis* ST3 contributes to reduced inflammation and accelerates recovery. In contrast, short-term colonisation has minimal effects on inflammation. The authors conclude that *Blastocystis*’ influence on intestinal health might be colonisation-dependent, hinting at potential protective effects in chronic settings (Kadlecová *et al*., ED P08). Moreover, innovative research explored drug discovery approaches against *Blastocystis* by leveraging methodologies used against pathogenic free-living amoebae (FLA). By applying anti-amoebic drug discovery techniques, this study targeted the amoeboid forms of *Blastocystis*, which may resemble those of FLAs (Sifaoui *et al*., ED T14).

Despite many studies investigating the association between *Blastocystis* presence and its clinical significance, the latter remains a topic of debate. Preliminary data from an ongoing project examining the associations of gallbladder removal surgery and the presence of *Blastocystis* STs, as well as how this influences microbiome composition and metabolite profiles, showed that several patients who were non-carriers before surgery became colonised post-surgery (Shaw *et al*., ED P08).

Addressing the ongoing debate on its pathogenic potential, a scoping review, adhering to PRISMA guidelines, aims to clarify the associations between *Blastocystis* infection and gastrointestinal health outcomes. This initiative aspires to fill knowledge gaps, potentially guiding future research in compliance with the COST action objectives (Tunali *et al*., ED T07).

## Diagnosis and molecular characterisation

The use of new diagnostic and subtyping tools for identifying and characterising *Blastocystis* is helping improve our understanding of its epidemiology, zoonotic potential, and transmission dynamics while also recognising patterns of ST host specificity ^39^. This was demonstrated using next-generation amplicon sequencing (NGS) in several works presented, a powerful tool enabling the identification of mixed infections in wild and domestic ungulates, including STs, at very low rates that would otherwise be unattainable when relying solely on Sanger sequencing ^40^. Patterns of cross-transmission between wild ungulate species (Dashti *et al*., ED P08) and herbivorous livestock (Figueiredo *et al*., ED T02) ^41^ were identified, along with up to twenty different STs—including zoonotic ones—in various populations sampled across Portugal and Spain. Both presentations showed distinctive ST host specificity across different wild and domestic ungulates, which was also demonstrated by a meta-analysis of *Blastocystis* ST distribution based on the analysis of *SSU* rRNA sequences from GenBank of various hosts and geographic regions (Sanda *et al*., ED T16). The finding that certain *Blastocystis* STs (or subgroups within STs) are specifically or preferentially found in particular host species opens the debate about whether intra-ST variability can play a role in the pathogenicity of the protist.

The application of NGS in human epidemiological studies was also shown in healthy children from Nicaragua, where four STs (ST1, ST2, ST3, and ST7) and a possible novel one were identified in mono- and mixed infections. The detection of ST7 suggests avian-human zoonotic transmission events; however, further genotyping analyses encompassing animal and environmental samples would be necessary to corroborate this finding (Comas-Murillo *et al*., ED P12). Another presentation demonstrated that *Blastocystis* infection rates and ST diversity were independent of the clinical status of patients with and without colorectal cancer in Colombia. NGS analyses identified eleven STs in mono- and mixed infections, comprising up to six different STs, with ST3 being the most commonly found (Hernández-Castro *et al*., ED P14). *Blastocystis* STs can be effectively identified with massive parallel amplicon sequencing using the Illumina MiSeq sequencing platform ^42^. However, this platform suffers from technical issues due to low heterogeneity in the *SSU* rDNA target region. To overcome this drawback, one presentation demonstrated that the addition of heterogeneity spacers can increase primer variation, preventing the same nucleotide from predominating in each sequencing cycle, which in turn can enhance signal diversity, efficiency, and quality of sequencing (Cinek *et al*., ED P23).

Applying NGS technology has enabled a deeper understanding of *Blastocystis* epidemiology; however, it remains a challenge in limited-resource settings. Recent advances in artificial intelligence (AI)-based microscopy, coupled with automated digital imaging, have allowed for the identification and quantification of parasitic developmental stages (including transmissive cysts) with high accuracy, improved quality, faster results, and a lower workload, thus contributing to better universal healthcare (El-Badry *et al*., ED T22). In fact, direct microscopy examination has proven to perform equally well as PCR-NGS in the detection of *Blastocystis* in endemic areas, displaying similar prevalence in patients with colorectal cancer (33.1 % *vs*. 34.6 %, respectively) (Hernández-Castro *et al*., ED P13). Nonetheless, both presentations emphasised the need for experienced parasitologists when relying on microscopy-based techniques.

The assorted presentations concerning human populations in different countries, including Albania (Abazaj *et al*., ED P24), Italy (Guadano Procesi *et al*., ED T03), Mexico (Juárez-Ramírez *et al*., ED T06), Nepal (Tabak *et al*., ED P18), the Republic of Kosovo and Lebanon (Pietrzak-Makyła *et al*., ED P04), Slovakia (Štrkolcova *et al*., ED P22), Slovenia (Jernej *et al*., ED T04), Spain (Goterris *et al*., ED P02), Tunisia (Akkari *et al*., ED P09), and Zambia (Mutengo *et al*., ED P01), as well as animal reservoirs including pet dogs in Slovenia (Jernej *et al*., ED T04), cattle in Tunisia (Akkari *et al*., ED P09), and captive non-human primates in Serbia (Petrovic *et al*., ED P16) and Slovakia (Kaduková *et al*., ED P19) have provided valuable insights into *Blastocystis* epidemiology and transmission dynamics contributing to the validation and standardisation of detection and ST characterisation methods while helping to clarify their public and animal health significance ^43^.

## Epidemiology and one health

The One Health approach is critical to understanding *Blastocystis*, as zoonotic STs are prevalent across diverse hosts and regions (Doğruman Al, ED T23). In a recently published review, high prevalence rates were reported worldwide: 41 % in Africa, 44 % in the Americas, 52 % in Australia and Oceania, and over 30 % in regions of Asia and Europe ^44^. *Blastocystis* transmission occurs through various environmental reservoirs, including water, soil, and fresh produce, highlighting the need for comprehensive control measures, such as improved sanitation and health education ^45^. The COST Action CA21105, " *Blastocystis* under One Health", leverages this approach, integrating medical, veterinary, public, and environmental health to bridge knowledge gaps, harmonise diagnostics, and enhance public health policies concerning *Blastocystis* infections ^46^. A study conducted in rural Algeria showed that *Blastocystis* (in addition to other enteric protist parasites) was a common finding in humans and cattle in close proximity (Al-Adilee et al., ED P10). It was also emphasised that there is a need for epidemiological studies targeting environmental samples, including water, fresh produce, and soil, as all these can act as reservoirs for the transmission of *Blastocystis* to humans and animals ^43^. Remarkably, a study with pre-washed vegetables from UK supermarkets identified *Blastocystis* ST1 and ST2 in 21% of the sampled products, showing that ready-to-eat vegetables can be a potential source of zoonotic transmission, underscoring the need for improved food safety practices to mitigate the risk of infection from environmental sources (Mavrides *et al*., ED P21).

Another topic discussed at the FIBC was that, since the available diagnostic tests have been optimised to detect the presence of the protist in faecal matter of human or animal origin, their use in other biospecimens would require adequate optimisation and validation to ensure correct diagnostic performance (El-Badry, ED T22). Finally, building trust in rural communities was identified as a key point when conducting One Health studies in remote areas of the world ^47^. Overall, studies under the One Health umbrella are expected to provide valuable data to better understand the epidemiology of *Blastocystis* and help unravel under which conditions it can behave as a pathogen or a beneficial symbiont.

## Future directions in *Blastocystis* research

The FIBC showcased *Blastocystis* and the breadth of research being undertaken to understand this protist from a One Health perspective. However, it also highlighted gaps in the field and areas of *Blastocystis* research that will need further attention.

Although the clinical significance of *Blastocystis* still remains uncertain, many researchers and physicians (particularly in the metagenomics field) are shifting towards the idea that it is beneficial to the host. It has become evident that there is a need for additional longitudinal studies to ascertain the long-term impact of *Blastocystis* colonisation on health and disease. It is important to note that with the advent of the expansion of longitudinal studies, it may be necessary to recruit state-of-the-art technologies to ensure consistency in sampling. Should *Blastocystis* be increasingly regarded as a beneficial symbiotic member of the gastrointestinal environment, what factors determine asymptomatic colonisation or symptomatic disease? Some bacteria can be referred to as pathobionts, implying that they are native to a host’s microbiome but can promote disease under certain conditions in the host, including gut dysbiosis ^48^. We question whether this should be a term used for microeukaryotes such as *Blastocystis* (and perhaps other species, including *Dientamoeba fragilis*), and whether certain microenvironmental factors are responsible for determining its behaviour.

In symptomatic patients, it is often difficult to determine the causative agent of the malady, with *Blastocystis* being stated as the aetiological agent only due to exclusion or lack of identification of any other potential pathogens. This also raises the question of whether we should exclude other diseases and symptoms when discussing *Blastocystis* pathogenicity. Properly designed case-control and randomised clinical trial studies would greatly assist in elucidating in which group of patients, and under which clinical conditions, *Blastocystis* can exert a pathogenic influence. There is also a need for more clinicians to include screening for *Blastocystis* in their routine diagnostic algorithms (either via microscopy or molecular techniques such as real-time PCR), as it is often overlooked and perhaps can have unforeseen roles as indicators or modulators of pathological processes.

As mentioned above, recent studies have started looking at metagenomic and microbiome studies of *Blastocystis* in the human host. Although such studies in animal hosts are still in their infancy, preliminary data presented at the FIBC support the idea that *Blastocystis* is a beneficial member of the gut microbiota in herbivores. Metagenomic studies are also needed to ascertain the link between *Blastocystis* colonisation and dietary profiles, explaining why strict carnivore species are less suited hosts for the protist than omnivore and strict herbivore species ^49^. In addition, further omics methods, such as proteomics for organelle-specific proteome mapping of *Blastocystis*, will benefit our understanding of this protist.

Since the introduction of the current subtyping nomenclature ^14^, a total of 44 distinct STs of *Blastocystis* have been formally described, and this number is expected to increase rapidly in the following years. This raises doubts about the practicality of the current classification scheme in the long term. Additionally, more research should be conducted to expand and integrate the taxonomy of *Blastocystis* in animal groups other than mammals and birds. New genetic markers and metagenomic tools will likely be needed to fulfil this task. One of the main problems associated with *Blastocystis* research is that axenic *in vitro* culturing is only available for a limited number of *Blastocystis* STs, including ST1, ST4 and ST7, thereby making it challenging to study its interactions with the host and other members of the gut microbiota. Axenic isolation would be essential to studying *Blastocystis* pathogenicity, describing its surface biochemical components and elucidating therapeutic effects. It is therefore important to expand the repertoire of available *in vitro* techniques to support the growth and investigation of *Blastocystis* in the laboratory. One such example could be through the use of microfluidic ‘gut-on-a-chip' devices, which aim to simulate a more physiologically relevant microenvironment than standard static culturing techniques.

Lastly, there is a need for more multidisciplinary collaborations between different research groups in the *Blastocystis* field, as well as further dissemination of any findings. The COST Action CA21105: *Blastocystis* under One Health facilitates these meetings and partnerships, as seen from this conference.

## Ethics and consent

Ethical approval and consent were not required.

## Data Availability

No data associated with this article. All data supporting the findings of this study are available within the paper and its Extended Data (available at https://zenodo.org/records/14576044 and doi: 10.5281/zenodo.14576043) ^50^. Data are available under the terms of the Creative Commons Attribution 4.0 International license (CC-BY 4.0) ( https://creativecommons.org/licenses/by/4.0/).
