# Supplementary material for: Update on Blastocystis: highlights from the Fourth International Blastocystis Conference
Source: Open Res Eur. 2025 Mar 7;5:11. Originally published 2025 Jan 20. [Version 2] doi: 10.12688/openreseurope.19168.2 (PMC11842961; doi:10.12688/openreseurope.19168.2)
Supplement: Peer review history

# Reviewer report: Reviewer response for version 1

**DOI:** 10.21956/openreseurope.20742.r50198

**Published:** 2025-02-20

**Authors:** Funda Dogruman-Al

**Affiliations:** 1 Gazi University, Ankara, Turkey

The reviewed article constitutes a comprehensive analysis of the 4th International Blastocystis Conference, sponsored by COST Action CA21105 “Blastocystis under One Health”, and the Microbiology Society, held in Crete (Greece) on 17-19 September 2024.

 It is seen that the authors explain the known and unknown about Blastocystis and emphasize the remarkable parts of the studies presented by participants from different disciplines on this subject.

 Especially in recent years, intestinal microbiome-based studies on humans regarding the relationship between Blastocystis and health and disease were included and it was emphasized that omics-based studies were needed in human and animals. The diversity of the participants made it possible to address Blastocystis with a one-health approach.

 However, it was stated that new studies were needed to elucidate the clinical importance of BLastocystis and its role in ecological balance, and that more in-depth information was required with developing technology.

 It was stated that the conference provided a productive environment in terms of the participation of young researchers and experienced researchers, the interactive sessions, the development of collaborations and the generation of new research ideas.

 It was observed that the article was a well-designed and systematic article that presented content, included detailed explanations with eye-catching titles and was written with proper and fluently English.

 Minor correction suggestions are given below.

 1. After the "Blastocystis background" section, where the term "subtype (ST)" is first used, it is appropriate to use only "ST" throughout the text.

 2. While the last paragraph of the "Diagnosis and molecular characterization" section mentions studies conducted in different countries, it should be ensured that all presentations related to this topic are included in the conference.

 3. 3. It is recommended that the "one health" approach presentations made on this topic at the conference be included in the "Epidemiology and One Health" section.

 Kind regards

Is the review written in accessible language?

Yes

Are all factual statements correct and adequately supported by citations?

Yes

Are the conclusions drawn appropriate in the context of the current research literature?

Yes

Is the topic of the review discussed comprehensively in the context of the current literature?

Yes

Reviewer Expertise:

Clinical Parasitology, Blastocystis, Gastrointestinal system protozoa, Microsporidia

I confirm that I have read this submission and believe that I have an appropriate level of expertise to confirm that it is of an acceptable scientific standard.

# Response

**Published:** 2025-03-02

**Authors:** David Carmena

**Affiliations:** Parasitology Reference and Research Laboratory, Instituto de Salud Carlos III, Majadahonda, Madird, Spain

***Reply to Prof. Funda Dogruman-Al´s comments*** *The reviewed article constitutes a comprehensive analysis of the 4th International Blastocystis Conference, sponsored by COST Action CA21105 “Blastocystis under One Health”, and the Microbiology Society, held in Crete (Greece) on 17-19 September 2024.* *It is seen that the authors explain the known and unknown about Blastocystis and emphasize the remarkable parts of the studies presented by participants from different disciplines on this subject.* *Especially in recent years, intestinal microbiome-based studies on humans regarding the relationship between Blastocystis and health and disease were included, and it was emphasized that omics-based studies were needed in human and animals. The diversity of the participants made it possible to address Blastocystis with a one-health approach.* *However, it was stated that new studies were needed to elucidate the clinical importance of Blastocystis and its role in ecological balance, and that more in-depth information was required with developing technology.* *It was stated that the conference provided a productive environment in terms of the participation of young researchers and experienced researchers, the interactive sessions, the development of collaborations and the generation of new research ideas.* *It was observed that the article was a well-designed and systematic article that presented content, included detailed explanations with eye-catching titles and was written with proper and fluently English. Minor correction suggestions are given below.*

*Reply: We thank the Reviewer (Prof. Funda Dogruman-Al) for her initial positive appraisal. All the comments and suggestions raised in her appraisal have been now included in the revised version of the manuscript.*  

1. *After the "Blastocystis background" section, where the term "subtype (ST)" is first used, it is appropriate to use only "ST" throughout the text.*

*Reply: The term “subtype” has been replaced by its abbreviation “ST” after being first mentioned in page 4 of the manuscript. See also our reply to comment #11 by Reviewer Barbara Soba on this specific matter.*  

1. *While the last paragraph of the "Diagnosis and molecular characterization" section mentions studies conducted in different countries, it should be ensured that all presentations related to this topic are included in the conference.*

*Reply: We have carefully revised all talk and poster presentations presented at the conference (see Extended Data available at https://zenodo.org/records/14576044 and doi: 10.5281/zenodo.14576043) and found that all of them related to the topic on this subsection have been adequately addressed. Please note that those considered more relevant have been commented in a more extensive way, whereas those providing more limited knowledge have been quoted as an assorted presentation.*  

1. *It is recommended that the "one health" approach presentations made on this topic at the conference be included in the "Epidemiology and One Health" section.*

*Reply: The following presentations have been now added in the Epidemiology and One Health subsection.:*

1.
  1. *Doğruman Al, ED T23.*
  2. *Al-Adilee et al., ED P10*

# Reviewer report: Reviewer response for version 1

**DOI:** 10.21956/openreseurope.20742.r50202

**Published:** 2025-02-18

**Authors:** Barbara Soba

**Affiliations:** 1 University of Ljubljana, Ljubljana, Slovenia

In the reviewed manuscript, the authors present the activities at the Fourth International Blastocystis Conference which took place in Heraklion (Crete, Greece) from 17 to 19 September 2024 and was mainly supported by the COST Action CA21105, “Blastocystis under One Health”, and the Microbiology Society. In the article, the authors first introduce the protist Blastocystis and then give a comprehensive description of the main topics discussed at the conference, from evolutionary biology and advances in Omics, intestinal ecology and clinical significance to diagnosis, molecular characterisation and epidemiology. At the same time, they present open questions that arise when studying this protist – whether its role is beneficial for the gut health of humans and animals or whether it acts as a pathogen, whether only certain subtypes might have pathogenic potential, etc. They propose research approaches that could help answer these questions.

 The authors aim to summarise current developments in Blastocystis research and to present the activities at the conference and within the COST action Blastocystis under One Health to the widest possible interested professional audience and thus encourage their collaboration.

 The topic of the manuscript is interesting and with some minor changes, 

 Abstract, Page 1, last Row: change “congress” to “conference”

 Abstract, Page 2, first Row: use double inverted commas as a quotation mark and move the comma, which is now in the quotation mark after the quotation mark

 Page 2, Plain language summary, Row 9: delete the space after the quotation mark

 Page 2, Plain language summary, Row 11: change “research” to “researchers”

 Page 2, Plain language summary, Row 17: change “congress” to “conference”

 Page 4, first Column, last Paragraph, Row 2: change “had” to “has”

 Page 4, first Column, last Paragraph, Row 7: change “Stenvold” to “Stensvold”

 Page 5, first Column, first Paragraph, Rows 8-12: “However, it was demonstrated that Blastocystis carriers are associated with the protist Entamoeba and the yeast Hanseniaspora, whereas non-carriers are associated with the intestinal hookworm Ancylostoma and the yeasts Malassezia, Candida, and Saccharomyces (Garzon et al., ED T01).” – this probably does not apply generally but to a specific geographic area. Please specify in the text the country where the study was conducted.

 Page 5, first Column, Paragraph 2, Row 20: “BMI” – explain the abbreviation

 Page 7, second Column, Paragraph 3, Row 10: The full stop after “species” is missing.

 Once you introduce the abbreviation ST for subtypes (Page 4), you can replace the word "subtype" with ST throughout the text. You do not need to introduce the abbreviation ST on Page 6 (first Column, last Paragraph) again.

Is the review written in accessible language?

Yes

Are all factual statements correct and adequately supported by citations?

Yes

Are the conclusions drawn appropriate in the context of the current research literature?

Yes

Is the topic of the review discussed comprehensively in the context of the current literature?

Yes

Reviewer Expertise:

parasitology, molecular diagnostics, epidemiology

I confirm that I have read this submission and believe that I have an appropriate level of expertise to confirm that it is of an acceptable scientific standard.

# Response

**Published:** 2025-02-19

**Authors:** David Carmena

**Affiliations:** Parasitology Reference and Research Laboratory, Instituto de Salud Carlos III, Majadahonda, Madird, Spain

**Reply to Dr Barbara Soba´s comments** The topic of the manuscript is interesting and with some minor changes. *Reply: We thank the Reviewer (Dr Barbara Soba) for her initial positive appraisal. All the comments and suggestions raised in her appraisal have been now included in the revised version of the manuscript.*  

1. Abstract, Page 1, last Row: change “congress” to “conference”

Reply: The term “congress” has been replaced by the term “conference” as per requested.  

1. Abstract, Page 2, first Row: use double inverted commas as a quotation mark and move the comma, which is now in the quotation mark after the quotation mark

Reply: Corrected as per requested.  

1. Page 2, Plain language summary, Row 9: delete the space after the quotation mark

Reply: Corrected as per requested (see the end of this document).  

1. Page 2, Plain language summary, Row 11: change “research” to “researchers”

Reply: Corrected as per requested (see the end of this document).  

1. Page 2, Plain language summary, Row 17: change “congress” to “conference”

Reply: Corrected as per requested (see the end of this document).  

1. Page 4, first Column, last Paragraph, Row 2: change “had” to “has”

Reply: The term “had” has been replaced by the term “has” as per requested.  

1. Page 4, first Column, last Paragraph, Row 7: change “Stenvold” to “Stensvold”

Reply: Corrected as per requested.  

1. Page 5, first Column, first Paragraph, Rows 8-12: “However, it was demonstrated that *Blastocystis* carriers are associated with the protist *Entamoeba* and the yeast *Hanseniaspora*, whereas non-carriers are associated with the intestinal hookworm *Ancylostoma* and the yeasts *Malassezia*, *Candida*, and *Saccharomyces* (Garzon et al., ED T01).” – this probably does not apply generally but to a specific geographic area. Please specify in the text the country where the study was conducted.

Reply: Thanks for your comment. To clarify this issue and improve readability we have rewritten the whole paragraph as follows: “However, a study conducted in a rural community in Colombia demonstrated that *Blastocystis* carriers were associated with the protist *Entamoeba* and the yeast *Hanseniaspora*, whereas non-carriers were associated with the intestinal hookworm *Ancylostoma* and the yeasts *Malassezia*, *Candida,* and *Saccharomyces. *Intriguingly, gut microbiota in Colombian *Blastocystis*-carriers populations mirrored Western Europeans, despite vastly diﬀerent environments and diets (Garzon *et al*., ED T01)”.  

1. Page 5, first Column, Paragraph 2, Row 20: “BMI” – explain the abbreviation

Reply: The abbreviation has been explained as follows: “body mass index (BMI)”.  

1. Page 7, second Column, Paragraph 3, Row 10: The full stop after “species” is missing.

Reply: Thanks for spotting this typo. Corrected as per requested.  

1. Once you introduce the abbreviation ST for subtypes (Page 4), you can replace the word "subtype" with ST throughout the text. You do not need to introduce the abbreviation ST on Page 6 (first Column, last Paragraph) again.

Reply: The term “subtype” has been replaced by its abbreviation “ST” after being first mentioned in page 4 of the manuscript. **Plain language summary:** *Blastocystis*, first identified in 1911, is one of the most common intestinal microorganisms found in humans; yet, little is known about its biology. Only recently have scientists started to investigate the role of *Blastocystis* in the gut microbiome and whether it causes disease in humans and animals. The Fourth International *Blastocystis *Conference, which was hosted in Heraklion (Crete, Greece) from the 17 ^th^ to the 19 ^th^ of September 2024, aimed to gather researchers from within the field to advance understanding and foster collaboration. The event was mainly supported by the COST Action CA21105, “ *Blastocystis* under One Health”, and the Microbiology Society. The event brought together researchers from various fields to discuss topics such as *Blastocystis’ *biology, its role in the gut microbiome, links to disease, methods of diagnosis, and its global impact. Presentations highlighted recent advancements in the fields, identified unanswered questions and outlined future research priorities. In this summary, we aim to provide a thorough overview of the presentations at the conference. The COST Action CA21105, *“Blastocystis* under One Health”, will build on the insights and collaborations formed during the conference, to advance our understanding of *Blastocystis *and better understand its implications for public health.
